# Enhanced long-term potentiation in the anterior cingulate cortex of tree shrew

**DOI:** 10.1098/rstb.2023.0240

**Published:** 2024-07-29

**Authors:** Qian Song, Xu-Hui Li, Jing-Shan Lu, Qi-Yu Chen, Ren-Hao Liu, Si-Bo Zhou, Min Zhuo

**Affiliations:** ^1^ Center for Neuron and Disease, Frontier Institutes of Science and Technology, Xi’an Jiaotong University, Xi’an 710049, People's Republic of China; ^2^ Neuroscience Research Center, Institute of Mitochondrial Biology and Medicine, Key Laboratory of Biomedical Information Engineering of Ministry of Education, School of Life Science and Technology and Core Facilities Sharing Platform, Xi’an Jiaotong University, Xi’an 710049, People's Republic of China; ^3^ Department of Physiology, Faculty of Medicine, University of Toronto, 1 King’s College Circle, Toronto, Ontario M5S 1A8, Canada; ^4^ Oujiang Laboratory (Zhejiang Lab for Regenerative Medicine, Vision and Brain Health), Wenzhou 325000, People's Republic of China; ^5^ Department of Neurology, First Affiliated Hospital of Guangzhou Medical University, Guangzhou 510030, People's Republic of China

**Keywords:** tree shrew, ACC, LTP, MED64, calcium imaging, non-human primate

## Abstract

Synaptic plasticity is a key cellular model for learning, memory and chronic pain. Most previous studies were carried out in rats and mice, and less is known about synaptic plasticity in non-human primates. In the present study, we used integrative experimental approaches to study long-term potentiation (LTP) in the anterior cingulate cortex (ACC) of adult tree shrews. We found that glutamate is the major excitatory transmitter and α-amino-3-hydroxy-5-methyl-4-isoxazole-propionicacid (AMPA) receptors mediate postsynaptic responses. LTP in tree shrews was greater than that in adult mice and lasted for at least 5 h. *N*-methyl-d-aspartic acid (NMDA) receptors, Ca^2+^ influx and adenylyl cyclase 1 (AC1) contributed to tree shrew LTP. Our results suggest that LTP is a major form of synaptic plasticity in the ACC of primate-like animals.

This article is part of a discussion meeting issue 'Long-term potentiation: 50 years on'.

## 1. Introduction

Synaptic plasticity is a well-studied phenomenon for regulating the intensity of synaptic transmission and is considered to be the cellular model for learning and memory, chronic pain, anxiety and fear [1–7]. However, most recent studies of long-term potentiation (LTP) have been carried out on rodents, while the information on LTP in primates is still scarce. Recent studies of LTP in monkeys have focused on imaging or behaviours [8,9], and less is known about cortical synaptic transmission and LTP [10]. Compared with rodents, the tree shrew (*Tupaia belangeri*) shares much more similar genome sequences with primates and is considered to have a close affinity to primates [11,12]. Considering its small body size, low cost of breeding and short reproductive cycle, the tree shrew provides a potentially useful animal model for investigating different physiological and pathological functions in primates (vision; spatial learning, stress and emotion adjustment) [13–19]. There are few studies of synaptic physiology and plasticity in the adult tree shrews.

The anterior cingulate cortex (ACC) is an important cortical brain area for regulating chronic pain, fear memory and anxiety [1–3,20–26]. In primates, ACC can be activated by noxious stimulation, pleasant touch, odours and taste [27–29]. In patients with frontal lobotomies or cingulotomies, the perception of pain and the ability to identify voice and facial expressions are abolished, accompanied by changes to social behaviour and subjectively experienced emotions [30,31]. LTP is considered to play a pivotal role in regulating these physiological and pathological processes. Controlling LTP in the ACC can reduce behavioural hyperalgesia [32]. Therefore, it is important to investigate the features of LTP in tree shrews.

In the present study, which aimed to reveal the character of cortical LTP in the tree shrew, we used a 64-channel multi-electrode dish (MED64) recording system, whole-cell patch recording and calcium imaging to record the LTP and the network property of LTP in the ACC of tree shrew. We found that theta burst stimulation (TBS) induced a long-lasting LTP in the ACC of adult tree shrews. The amplitude of LTP in the adult tree shrew is greater than that of adult mice. Similar to the ACC of adult mice, *N*-methyl-d-aspartic acid (NMDA) receptors, especially the GluN2B receptor, and L-type Ca^2+^ channels, are important for excitatory synaptic transmission.

## 2. Methods

### (a) Animals

Experiments were performed with adult male tree shrews (purchased from Kunming Institute of Zoology) and C57BL/6 J mice. All tree shrews and mice were maintained on a 12 h light/dark cycle with food and water provided ad libitum. All experimental protocols were approved by the Animal Care and Use Committee of the University of Xi’an Jiaotong University.

### (b) Slice preparation

The general methods for preparing ACC slices of tree shrew were similar to those previously described for mice [23,33,34]. Adult male tree shrews and mice were anaesthetized with isoflurane, the whole brain was quickly removed from the skull and submerged in ice-cold, oxygenated (95% O_2_ and 5% CO_2_) cutting solution containing (in mM) 252 sucrose, 2.5 KCl, 6 MgSO_4_, 0.5 CaCl_2_, 25 NaHCO_3_, 1.2 NaH_2_PO_4_ and 10 glucose, pH 7.3–7.4. After cooling in the cutting solution for a few minutes, the whole brain was trimmed to create an appropriate plane to glue onto the ice-cold platform of a vibrating tissue slicer (VT1200S, Leica). Coronal brain slices (300 μM), containing the ACC, were prepared after the corpus callosum connected. After cutting, slices were then incubated in a submerged recovery chamber with the artificial cerebrospinal fluid (ACSF) containing the following (in mM): 124 NaCl, 4.4 KCl, 2 CaCl_2_, 1 MgSO_4_, 25 NaHCO_3_, 1 NaH_2_PO_4_ and 10 glucose, pH 7.3–7.4, for at least 1 h at room temperature.

### (c) Multi-channel field potential recordings

After incubation for 1 h at room temperature, one slice was positioned on the MED64 probe and the ACC area was entirely covered by the recording dish, which was mounted on the stage of an inverted microscope (CKX41, Olympus). A fine mesh anchor (Warner Instruments, Harvard) was carefully positioned on the settled slice to ensure slice stability during recording. The slice was continuously perfused with oxygenated ACSF, 2 ml min^–1^ with the aid of a peristaltic pump (Minipuls 3, Gilson) throughout the experimental period.

During the recording period, one of the channels located in the deep layer (V–VI) of the ACC was chosen as the stimulation site. Monopolar and biphasic constant current pulses (10–20 μA, 0.2 ms) were applied to the stimulation site and field excitatory postsynaptic potentials (fEPSPs) evoked at a both superficial layer (II–III) and a deep layer (V–VI) of the ACC using MED Mobius software. The fEPSP was displayed on the monitor screen, amplified by a 64-channel amplifier and stored on the microcomputer for analysis. After the baseline was stabilized for 1 h, a weak TBS protocol (five bursts at 5 Hz, four pulses at 100 Hz for each burst) was given once time or a TBS protocol (five bursts at 5 Hz, four pulses at 100 Hz for each burst) was given five times (10 s interval) to induce post-LTP at the stimulation intensity, which was adjusted to elicit 40–60% of the maximal response [23,33]. To test the compound of LTP, an NMDA receptor antagonist (AP5, 100 μM, 60 min), L-type Ca^2+^ channel blocker (Nimodipine, 30 μM, 60 min) and adenylyl cyclase 1 (AC1) antagonist (NB001, 1 μM, 60 min) were applied 30 min before and after the weak TBS or TBS protocol, respectively. The synaptic responses were monitored for 5 h after stimulation or drug application to see the time course of post-LTP. The percentages of the fEPSP slopes were normalized by the averaged value of the baseline. A channel was denoted as displaying LTP if the response was increased by at least 15% of the baseline during this period.

### (d) Two-photon calcium imaging

*In vitro* calcium imaging was performed using a two-photon laser scanning microscope (Olympus FV1000-MPE system, BX61WI microscope) based on a pulsed Ti-sapphire laser (MaiTai HP DeepSee, 690–1040 nm wavelength, 2.5 W average power, 100 fs pulse width, 80 MHz repetition rate; New Port Spectra-Physics, Santa Clara, CA, USA). The laser was focused through a ×40 water-immersion objective lens (LUMPLFL/IR40XW, N.A.: 0.8, Olympus) and the average power was set to <15 mW (measured under the objective). Neurons were filled with indicators via the patch pipette for 20–30 min to allow diffusion of the dye into the cells. Fluorescent imaging of Cal-520 K^+^ salt (200 μM) and Alexa594 K^+^ salt (20 μM) was separated into green and red channels by a dichroic mirror and emission filters (Chroma, Bellows Falls, VT, USA) and detected by a pair of photomultiplier tubes (Hamamatsu, Shizuoka, Japan) at 800 nm. To obtain time series of fluorescent signals from global soma images, images were collected with the following parameters [35–38]: 512 × 512-pixel images, digital zoom 3× with ×40 objective (N.A. 0.8), 2-μs pixel dwell time, 50 ms/frame for frame scan model with different recording times for different recording frames. Bidirectional scanning and line-scanning models were used to increase scan speed. Each trial was repeated at least three times and the mean value was collected. Fluorescence changes were quantified as increases in green fluorescence from the baseline of Δ*F*/*F* = (*F − F_0_*)/*F_0_*.

**Figure 1.**
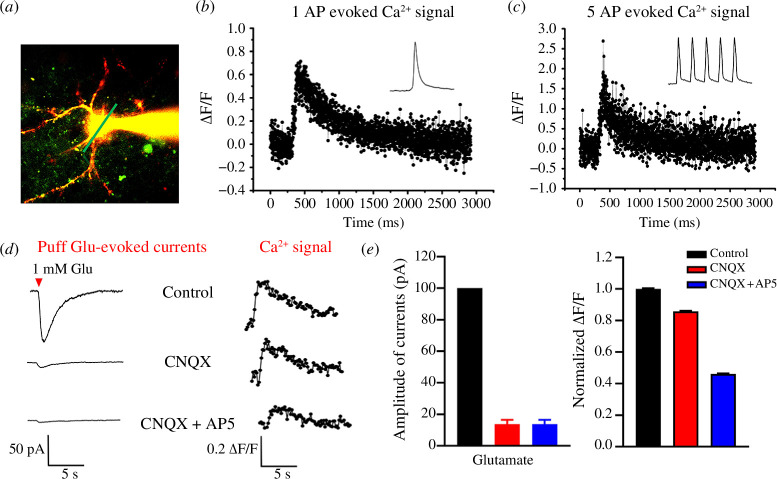
Action potential (AP) and puffing-Glu-induced calcium imaging in the ACC of tree shrew. (*a*) Representative two-photon fluorescent image of patch neuron loading by Alexa 594 and Cal-520 K^+^ salt. (*b* and *c*) Single AP (*b*) and 5 APs (*c*) evoked Ca^2+^ signals in soma. Waveforms of fluorescence change (Δ*F*/*F*) in response to a single AP (*b*) and 5 APs (*c*) in soma. (*d*) Average traces of puff application of Glu-evoked currents (left) and associated Ca^2+^ signals (Δ*F*/*F*) (right) evoked by puff-application of 1 mM Glu (10 psi, 100 ms, *n* = 6 neurons from 3 mice). (*e*) Summary results showing the percentage of application of Glu-evoked currents (left) and Ca^2+^ signals (Δ*F*/*F*, right) in the presence of CNQX (25 μM) and ᴅ-2-amino-5-phosphonovalerate (AP5) (50 μM). Arrows in (*a–e*) indicate a starting point of the pairing protocol application. Error bars in (*e*) represent s.e.m.

In figure 1, a picopump (WPI Pneumatic PicoPump, Sarasota, FL) was used for puff application of glutamate (Glu, 1 mM). Before establishing whole-cell recording, the drug application pipette was moved beside the neuron using a micromanipulator (Sutter MP-285, Novato, CA). The tip of the pipette was 50 µm away from the recorded neuron. The diameter of the drug application pipette tip was 3–4 µm. The pressure and duration of the puff were 15 psi and 100 ms, respectively [35]. 6-Cyano-7-nitroquinoxaline-2,3-dione (CNQX) and ᴅ-2-amino-5-phosphonovalerate (AP5) were applied to test the contribution of α-amino-3-hydroxy-5-methyl-4-isoxazole-propionicacid (AMPA) receptors receptorsand *N*-methyl-d-aspartic acid (NMDA) receptors on the puff application of Glu-induced calcium imaging in the tree shrew neurons.

### (e) *In vitro* whole-cell patch-clamp recording

Experiments were performed in a recording chamber using an Olympus BX51W1 microscope with infrared differential interference contrast (DIC) optics for the visualization of whole-cell patch clamp recording. In the present study, evoked excitatory post-synaptic currents (eEPSCs) were recorded from the layer II/III neurons with an Axopatch 200B amplifier (Molecular Devices, CA) and the stimulations were delivered by a bipolar tungsten stimulating electrode placed in the layer V/VI of the ACC slices. Control test pulses were given every 30 s. The amplitudes of eEPSCs were adjusted to between 50 and 100 pA to obtain a baseline. The recording pipettes (pyramidal neurons, 3–5 MΩ) were filled with a solution containing 145 mM K-gluconate, 5 mM NaCl, 1 mM MgCl_2_, 0.2 mM ethylene glycol tetraacetic acid (EGTA), 10 mM 4-(2-hydroxyethyl)-1-piperazineethane sulfonic acid (HEPES), 2 mM Mg-adenosine triphosphate (ATP) and 0.1 mM Na_3_-guanosine triphosphate (GTP) (adjusted to pH 7.2 with KOH, 290 mOsmol). Picrotoxin (100 μM) was always present to block γ-aminobutyric acid_A_ (GABA_A_) receptor-mediated inhibitory synaptic currents in all experiments. The membrane potential was held at −60 mV for eEPSCs recordings. To examine synaptic responses, the input (stimulus intensity)–output (EPSC amplitude) (I–O curves) relationships in the ACC pyramidal neurons were recorded at different stimulus intensities. NMDAR-mediated EPSCs were recorded at −20 mV by bathing with 6-cyano-7-nitroquinoxaline-2,3-dione (CNQX: 25 μM). The patch electrode internal solution (in mM) 112 Cs-gluconate, 5 tetraethylammonium chloride (TEA-Cl), 3.7 NaCl, 0.2 EGTA, 10 HEPES, 2 Mg-ATP, 0.1 Na_3_-GTP and 5 QX-314 (adjusted to pH 7.2 with CsOH, 290 mOsmol) was used for recording NMDA receptor-mediated EPSCs. The access resistance was 15–30 MΩ and was monitored throughout the experiment. Data were discarded if the access resistance changed by more than 15% during the experiment. Data were filtered at 1 kHz and digitized at 10 kHz.

### (f) Tissue preparation and western blot analysis

Subcellular fractionation of tissue was prepared as described previously [39]. Several brain regions: prefrontal cortex (PFC), ACC, insular cortex (IC), primary somatosensory cortex (S1), hippocampus, motor cortex (M) and spinal cord dorsal horn (SC.d) were dissected on ice in cold ACSF and homogenized in 0.32 M sucrose buffer containing 10 mM HEPES (pH 7.4), a protease inhibitor and phosphatase inhibitor cocktails, and then centrifuged at 4°C, 1000 *g* for 10 min to yield the nuclear-enriched pellet and the S1 fraction. The S1 fraction was then centrifuged (12 000 *g*, 20 min, 4°C) to obtain the pellet (P2; crude synaptosomal membranes) fraction and supernatant S2. The synaptosomal pellet P2 was resuspended in 4 mM HEPES buffer 4 mM HEPES buffer (pH 7.4, 1 mM ethylene diamine tetraacetic acid (EDTA)) and again centrifuged (12 000 *g*, 20 min, 4°C). The pellet was resuspended in buffer A (20 mM HEPES buffer, pH 7.2, 100 mM NaCl, 0.5% Triton X-1000) and rotated slowly for 15 min, 4°C, followed by centrifugation (12 000 *g*, 20 min, 4°C). The supernatant was obtained as non-postsynaptic density (PSD) fraction. The pellet was resuspended in buffer B (20 mM HEPES buffer, pH 7.5, 0.15 mM NaCl, 1% Triton X-100, 1% deoxycholic acid, 1% sodium dodecyl sulfate (SDS) and 1 mM dithiothreitol (DTT). The supernatant was obtained as PSD fraction. Fraction samples were characterized by western blot or stored at −80°C.

Western blotting was performed as reported previously [39]. Protein concentrations were determined using Bio-Rad protein reagent. Equal amounts of protein were subjected to sodium dodecyl sulfate–polyacrylamide gel electrophoresis (SDS–PAGE) and then transferred onto polyvinylidene membranes at 4°C. After blocking 5% milk in tris-buffered saline Tween-20 (TBS-T) buffer, the membranes were incubated with primary antibodies GluN1 (05–432, 1:500, Millipore), GluN2A (AB1555, 1:2000, Millipore), GluN2B (AB1557, 1:1000, Millipore), and Tubulin (T5201, 1:5000, Sigma) overnight at 4°C. After washing in TBS-T, the membranes were incubated with a secondary antibody (1:5000) for 1 h, followed by enhanced chemiluminescence detection of the proteins with the Western Lightning Chemiluminescence Reagent Plus. ImageJ software was used to assess the density of the immunoblots by a blind observer.

### (g) Drugs and antibodies

The chemicals, drugs and antibodies used in this study were as follows:

CNQX was purchased from Sigma-Aldrich. d-(-)-2-amino-5-phosphonopentanoic acid (AP5), Nimodipine, NB001, PEAQX and Ro256981 were purchased from Tocris Cookson (Bristol, UK). Cal-520 K^+^ salt and Alexa594 K^+^ salt were purchased from AAT Bioquest. Drugs were prepared as stock solutions for frozen aliquots at −20°C. All these drugs were diluted from the stock solution to the final desired concentration in the ACSF before immediate use. Antibodies against GluN1 (05–432), GluR2A (AB1555), and GluN2B (AB1557) were purchased from Millipore, Tubulin (T5201) was purchased from Sigma. Horseradish peroxidase (HRP)-linked goat anti-mouse IgG (AP308P, 1:5000）and goat anti-rabbit IgG (AP307P, 1:5000) were both purchased from Millipore.

### (h) Data analysis

Results are expressed as mean ± s.e.m. Statistical analyses were conducted using GraphPad Prism (GraphPad Software). Unpaired Student’s *t*‐test was used for statistical comparisons. The level of significance was set at *p < 0.05.

## 3. Results

### (a) AMPA receptor mediates basal synaptic transmission in the ACC of tree shrew

**Figure 2.**
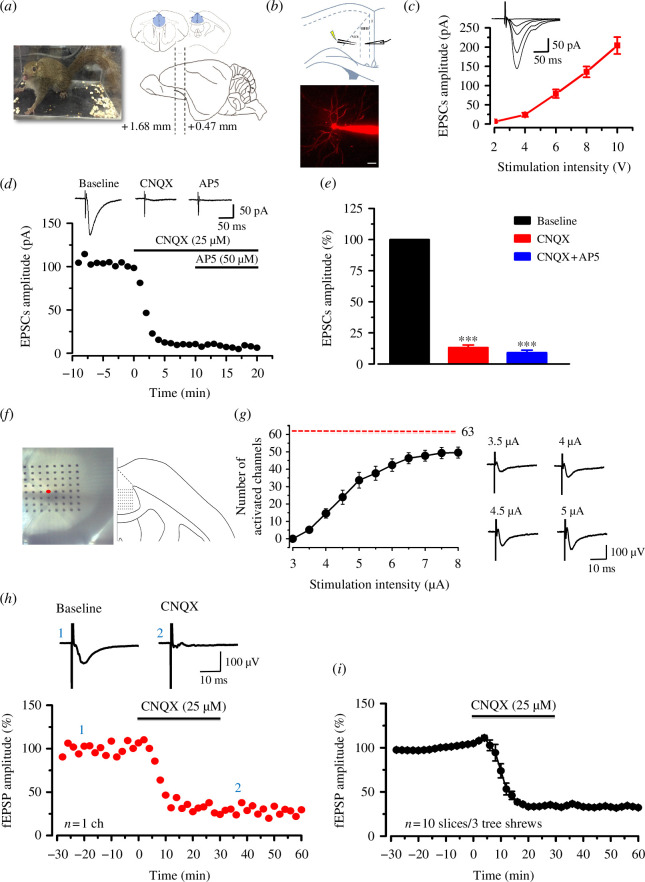
Glutamate receptor-mediated synaptic transmission in the ACC of tree shrew. (*a*) The photo shows the process of anaesthesia of tree shrew with 1–2% isoflurane (left). The schematic diagram showed slices including ACC area (from Bregma +1.68 to +0.47 mm) of tree shrew was used for the research (right). (*b*) Schematic diagram showing the placement of stimulating and recording electrodes in the ACC of tree shrew (top). Representative photomicrograph of an Alexa 594-labelled pyramidal neuron in layer II/III of ACC (bottom). Scale bar: 50 μm. (*c*) Sample traces and pooled data show the input–output relationship of basal EPSCs in the ACC of tree shrew (*n* = 8 neurons/4 tree shrews). (*d*) Sample time course points show the EPSCs in the presence of CNQX and AP5. EPSCs were recorded in the presence of picrotoxin (100 μM). After the perfusion of CNQX (25 μM) for 10 min, a small residual current remained that could be blocked by CNQX and AP5 (50 μM) together. (*e*) Statistical results show the percentage of EPSCs in the presence of CNQX and AP5 (*n* = 8 neurons/4 tree shrews). Insets in (*d*) are sample traces with the perfusion of CNQX and AP5, respectively. (*f*) The microphotograph and schematic diagram show one example of ACC fEPSP recording by using the MED64 system. A cortex slice containing the ACC of tree shrew was placed on a probe (MEDP515A, 8 × 8 array). One channel of the probe (red circle) was selected as the stimulation site. The evoked field potentials in all the other 63 channels were recorded 1 h before and 5 h after TBS. (*g*) The number of activated channels induced by different stimulation intensities (input–output) in tree shrew (left) and in sample traces (right) show one channel with enhanced fEPSP in response to different intensities of stimuli in tree shrew. (*h*) The fEPSP slope and the sample traces from one channel show that CNQX blocked the potential. (*i*) The summarized fEPSP slopes show that CNQX blocked all activated potentials from 10 slices in 3 tree shrews. Calibration: 100 µV, 10 ms. Error bars in (*c*), (*e*) and (*i*) represent s.e.m. Unpaired Student’s *t*‐test, ***p < 0.001.

We used the whole-cell patch *in vitro* to test the characteristics of synaptic transmission in the ACC of tree shrew. The EPSCs were recorded in pyramidal neurons in layer II/III of ACC using Alexa594-labelled recording electrodes (figure 2*a*,*b*). The input (stimulation intensity)–output (EPSCs amplitude) (I–O curves) were recorded in ACC neurons of tree shrews (figure 2*c*, *n* = 8 neurons/4 tree shrews). The EPSCs were recorded stabilized for at least 10 mins as the baseline. After bath application of the CNQX (25 µM) for 10 min, the EPSCs were reduced severely and a small residual current remained as 13.2 ± 2.1% of baseline that could be blocked as 9.1 ± 2.0% of baseline by application of CNQX and AP5 (50 µM) together (figure 2*d,e*). These results indicate that the synaptic transmission in the ACC was mediated by glutamate AMPA/kainate receptors.

To characterize whether there is a similar neuronal network connection, we used the MED64 recording system to map the cortical circuit responses within the ACC of tree shrew according to focal electrical stimulation (figure 2*f*). One channel in the deep layer of the ACC (layer V) was then chosen as the stimulation site, and the other 63 channels were used for measuring evoked responses. We found that local stimulation induced widespread fEPSPs, which could be observed both in superficial and deep layers and concentrated at the stimulation site. The number of channels with evoked fEPSPs (active channels) was dependent on the intensities of stimulation and reached a maximum of 49.6 ± 3.1 (*n* = 12 slices/6 tree shrews; figure 2*g*). After bath application of the AMPA/kainate receptor antagonist CNQX (25 µM), the fEPSPs of one channel, one slice and all 10 slices from 3 tree shrews were almost abolished (figure 2*h,i*). These results also showed that the synaptic transmission in the ACC was mediated mostly by glutamate AMPA/kainate receptors.

### (b) NMDAR-mediated synaptic transmission in the ACC of tree shrew

**Figure 3.**
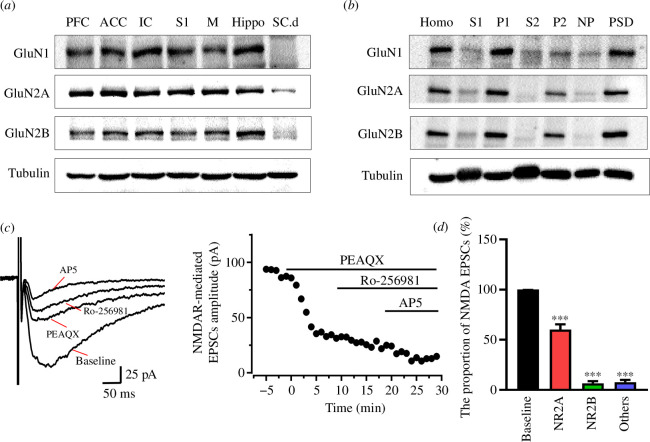
GluN2B receptor-mediated synaptic transmission in the ACC of tree shrew. (*a*) Representative western blots for GluN1, GluN2A and GluN2B in different brain areas and SC.d of tree shrew. (*b*) Subcellular localization of GluN1, GluN2A and GluN2B in the ACC of tree shrew. (*c*) Time course of the amplitudes of NMDA receptors-mediated eEPSCs with application of PEAQX, Ro-256981 and AP5 in the ACC of tree shrew. (*d*) The summarized proportions of NMDA eEPSCs with PEAQX, Ro-256981 and AP5 application. Unpaired Student’s *t*‐test, ***p < 0.001. Error bars in (*d*) represent s.e.m.

Considering that the NMDAR is critical for synaptic transmission and LTP, we then tested the composition of the NMDAR subtype that was measured in the tree shrew. By using western blot, we found that GluN1, GluN2A and GluN2B subtypes can be found in the ACC, as well as in the PFC, insular cortex (IC), primary somatosensory cortex (S1), motor cortex (M), hippocampus (Hippo) and SC.d (figure 3*a*). Using subcellular fractionation of the ACC, we found that GluN1, GluN2A and GluN2B receptors were located at the synaptic sites of the ACC (figure 3*b*). In addition, the GluN2A and GluN2B receptor-mediated EPSCs were measured in the ACC neuron of tree shrew by performing whole-cell patch recording. As shown in figure 3*c,d*, the NMDAR-mediated EPSCs were significantly blocked by bath application of GluN2A antagonist PEAQX (1 µM) and continued reduced by application of GluN2B antagonist Ro-256981 (3 µM) (*n* = 6 neurons/3 tree shrews). The GluN2A antagonist reduced around 60.2 ± 5.2% of NMDAR-mediated EPSCs, and the application of GluN2B antagonist further decreased the EPSCs to around 6.7 ± 2.1%. It is worth noting that a small residual current was exited even after AP5 was applied, suggesting AP5 may not be enough to block the NMDAR-mediated EPSCs in the tree shrew neurons.

Considering that the NMDA receptor-dependent Ca^2+^ signal is critical for synaptic transmission and LTP, we then tested the global Ca^2+^ signal by combining whole-cell patch recording and two-photon Ca^2+^ imaging in the ACC pyramidal neurons of tree shrew. As shown in figure 1*a*, the neuronal morphology was well labelled by Alexa594 and Cal-520 K^+^ salt after 30 min diffusion in the recording pipette. We found that global calcium transients were clearly observed when single action potentials (APs) occurred, which could be induced by injecting depolarizing currents into the soma of cells (figure 1*b*). The Δ*F*/*F* values of Ca^2+^ signals were significantly increased in an intensity- and frequency-dependent manners (five APs at 20 Hz; figure 1*c*). Next, we tested Ca^2+^ influx evoked in the tree shrew neuron by the puff-application of exogenous glutamate (Glu, 1 mM). As shown in figure 1*d,e*, puff-application of Glu-evoked currents was significantly reduced by AMPA receptor antagonist CNQX and almost completely blocked by additional NMDA receptor antagonist AP5, while puff-application of Glu was associated with a remarkable subthreshold soma calcium influx, and only a small reduction by CNQX even though puff-application of Glu-evoked currents was almost inhibited. The application of AP5 significantly reduced the Ca^2+^ signals. These results suggest that the NMDA receptor in the ACC neuron of tree shrew is a major contributor to the Ca^2+^ signal, with the residual Ca^2+^ signal possibly mediated by voltage-gated calcium channels or the calcium-permeable AMPA receptor.

### (c) Multi-channel recordings of TBS and weak TBS-induced LTP in the ACC circuit of tree shrew

**Figure 4.**
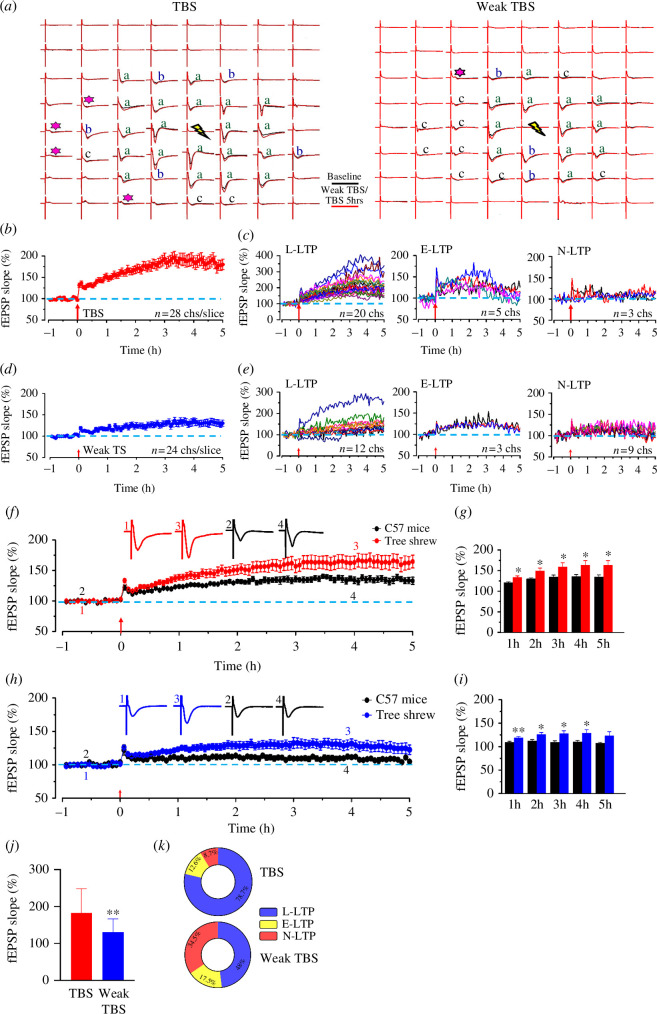
Multi-channel recordings of post-LTP in the adult tree shrew ACC. (*a*) The mapped samples show the network fEPSP in the ACC of tree shrew with TBS (left) and weak TBS (right). The fEPSP was induced by electrical stimulation on one channel (37, marked as yellow flash) and was recorded from the other 63 channels 1 h before (black) and 5 h after (red) TBS or weak TBS was delivered to one channel marked as yellow flash. Asterisks from the mapped figure of tree shrew indicate the channels with recruited fEPSP. Three types of plasticity are also shown in the mapped samples: channels showing typical late-phase LTP (L-LTP) are marked as **a**, channels showing relative short-term potentiation after TBS are marked as **b**; channels without potentiation in the slope of the fEPSP are marked as **c**. (*b*) The final averaged slope for all 28 active channels was 182.5 ± 13.1% of the baseline at 5 h after TBS stimulation. (*c*) The sample slope of three types of plasticity from 1 slice of 1 tree shrew. The fEPSP slopes of 20 channels with L-LTP (left), 5 channels with short-term potentiation (early-phase LTP, E-LTP, middle) and 3 channels without potentiation (N-LTP, right) are shown, respectively. (*d*) The final averaged slope for all 24 active channels was 130.9 ± 7.6% of the baseline at 5 h after weak TBS stimulation. (*e*) The sample slopes of three types of plasticity from 1 slice of 1 tree shrew. The fEPSP slopes of 12 channels with L-LTP (left), 3 channels with short-term potentiation (middle) and 9 channels without potentiation (right) are shown, respectively. (*f*) Time course of the averaged fEPSP slopes of all recorded channels in C57 mice and tree shrew (8 slices/4 mice and 11 slices/5 tree shrews in each group) with TBS. (*g*) The averaged slope of fEPSP from total active channels of C57 mice and tree shrew at different time points after the TBS stimulation. (*h*) Time course of the averaged fEPSP slope of all recorded channels in C57 mice and tree shrew (6 slices/4 mice and 9 slices/3 tree shrew in each group) with weak TBS. The mean percentage of fEPSP slope was potentiated to 108.8 ± 2.7% of the baseline in mice and 127.1 ± 9.0% of baseline at the end of the recording period (*p* < 0.001, paired *t*‐test). (*i*) The averaged slope of fEPSP from total active channels of C57 mice and tree shrew at different time points after the weak TBS stimulation. (*j*) Statistical results of the averaged slope of fEPSP with TBS and weak TBS. (*k*) Pie graphs summarizing the three types of plasticity from tree shrew with TBS (top) and weak TBS (down). Error bars in (*b*, *d*, *f–j*) indicate s.e.m. Unpaired Student’s *t*‐test, *p < 0.05, **p < 0.01.

In previous studies, by applying a multi-channel recording system, we have tested the intercellular connections in the ACC of mice [23,40]. To characterize the possibility of L-LTP induction within the ACC of tree shrew, we then applied TBS (five trains of burst with four pulses at 100 Hz, at 200 ms intervals; figure 4*a*) to induce LTP in the stimulation site in the deep layer of ACC from tree shrew, after 1 h baseline recording. When comparing with mice, both the occurrence ratio and degree of potentiation were larger in tree shrew. In one typical sample slice of tree shrew with 28 active channels, 20 channels showed potentiation of the slope lasting for 5 h (212.0 ± 12.4% of the baseline), 5 channels showed short-term potentiation and 3 channels remained stable throughout the entire recording period (figure 4*c*). The final averaged slope for all 28 active channels was 182.5 ± 13.1% of the baseline at 5 h after TBS stimulation (figure 4*b*). In total, in 112 active channels (averaged 14.0 ±1.7%) from 8 slices/4 mice,82 channels (73.2 ± 1.2%) showed L-LTP that lasted for 5 h, 13 channels (11.6 ± 1.9%) showed short-term potentiation and 17 channels (15.2 ± 1.8%) showed no potentiation. In total, in 230 active channels from 11 slices/5 tree shrews, 181 channels (78.7 ± 1.2%) showed L-LTP, 29 channels (12.6 ± 2.0%) showed short-term potentiation and 20 channels (8.7 ± 0.4%) showed no potentiation (figure 4*k*). Meanwhile, the TBS-induced potentiation degree was larger in tree shrew than in C57 mice at different time points after L-LTP induction (table 1, *p* < 0.05 in each time point, figure 4*f,g*).

**Table 1.** Multi-channel recordings of TBS-induced LTP in the ACC of tree shrew and mice.

	1 h after TBS	2 h after TBS	3 h after TBS	4 h after TBS	5 h after TBS
mice	121.1 ± 1.8%	130.1 ± 2.3%	134.9 ± 4.5%	135.8 ± 4.4%	134.5 ± 5.0%
tree shrew	133.3 ± 4.1%	149.1 ± 7.0%	159.5 ± 9.8%	163.2 ± 10.8%	163.3 ± 11.1%

It has been proved that weak TBS activates relatively few afferent fibers, and cannot trigger LTP in the hippocampus in mice [41]. We thus wanted to test if weak TBS could induce the LTP in the ACC of tree shrews. After 1 h baseline recording, weak TBS (five trains of burst with four pulses at 100 Hz, at 200 ms intervals) was applied in the stimulation site in the deep layer of ACC from tree shrew and mice. It was found that the fEPSPs of most of the active channels (50.5 ± 2.1% from 6 slices/4 mice) could not be potentiated in mice. It was found that the fEPSPs of most of the active channels (65.5%) could be potentiated in tree shrews. Interestingly, around half of the active channels (48%) showed L-LTP of the fEPSPs in tree shrew and the slope of fEPSP in one typical channel was enhanced to 140.7 ± 7.5% of the baseline. In one sample slice of tree shrew with 24 active responses, 12 showed potentiation of the fEPSP slope lasting for 5 h (152.8 ± 11.8% of the baseline), 3 showed short-term potentiation and 9 remained stable throughout the entire recording period (figure 4*e*). The final averaged slope for all 24 active channels was 129.9 ± 1.6% of the baseline at 5 h after weak TBS stimulation (figure 4*d*). In total, 109 active channels (averaged 18.2 ± 2.9%) from 6 slices/4 mice, 41 channels (37.6 ± 2.3%) showed L-LTP that lasted for 5 h, 13 channels (11.9 ± 2.8%) showed short-term potentiation and 55 channels (50.5 ± 2.1%) showed no potentiation. In total, in 200 active channels from 9 slices/5 tree shrews, 96 channels (48 ± 3.0%) showed L-LTP, 35 channels (17.5 ± 1.5%) showed short-term potentiation and 69 channels (34.5 ± 2.0%) showed no potentiation (figure 4*k*). Meanwhile, the weak TBS-induced potentiation degree was larger in tree shrew than in C57 mice at different time points after L-LTP induction (table 2, *p* < 0.01 in 1 h after TBS, *p* < 0.05 in other time point, figure 4*h*,*i*). The results showed that the weak TBS can induce the L-LTP in tree shrew, but the slope of the fEPSP potentiation degree was lower than TBS in tree shrew (figure 4*j*).

**Table 2.** Multi-channel recordings of weak TBS-induced LTP in the ACC of tree shrew.

	1 h after weak TBS	2 h after weak TBS	3 h after weak TBS	4 h after weak TBS	5 h after weak TBS
mice	109.7 ± 2.1%	112.3 ± 2.7%	109.5 ± 2.6%	110.5 ± 2.8%	108.8 ± 2.7%
tree shrew	119.9 ± 2.4%	126.4 ± 5.0%	129.4 ± 7.1%	131.2 ± 7.9%	127.1 ± 9.0%

### (d) Recruitment of synaptic responses within the ACC network after TBS and weak TBS induction

**Figure 5.**
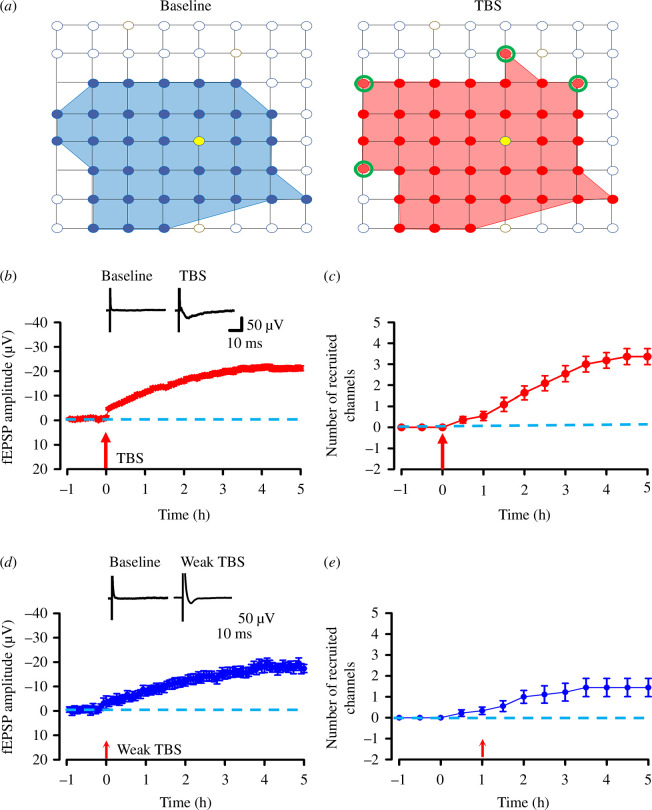
Spatial analysis of post-LTP distribution in the ACC of tree shrew. (*a*) Polygonal diagram of the channels that are activated in the baseline (blue, left) and after TBS (red, right) in tree shrew. The blue and red circles and regions denote activated channels. The green circles indicate the recruitment channels in the slice. The stimulation sites are marked as yellow circles. (*b*)Summary of the typical trace and the amplitude of fEPSP were summarized from all recruited channels (*n* = 16 channels from 9 slices/4 tree shrews). (*c*) Summary of the number of recruited channels was summarized after TBS induction. (*d*) The typical trace and the summary graph of the amplitude of fEPSP of recruited channels (*n* = 13 channels from 9 slices/3 tree shrews) from tree shrew with weak TBS. (*e*) Summary or the number of recruited channels was summarized after weak TBS induction. Arrows in (*b–e*) indicate starting point of TBS or weak TBS application. Error bars in (*c*, *e*) represent s.e.m.

The MED64 recording system provides a convenient way to study the cortical network L-LTP. The distribution of all activated channels during the whole recording was displayed by a polygonal graph (the blue lines represent the activated channels during the baseline and the red lines represent the activated channels after TBS or weak TBS). In our previous studies from mice, we have confirmed that some channels that were inactive during baseline recordings showed evoked fEPSPs after TBS induction (recruited channels) [23]. From our research, we found that both TBS and weak TBS can induce the recruited responses in tree shrew (figure 5*a*). After TBS or weak TBS induction, the average amplitude of fEPSPs of recruited channels gradually increased (finally reached as large as −21.2 ± 1.0 μV with TBS and −18.2 ± 1.7 μV with weak TBS) and remained stable for 5 h (figure 5*b,d*). At 5 h after TBS or weak TBS, the number of recruited channels reached 3.4 ± 0.4 with TBS (*n* = 37 channels from total 11 slices/5 tree shrews, figure 5*c*) and 1.4 ± 0.4 with weak TBS (*n* = 13 channels from total 9 slices/3 tree shrews, figure 5*e*). Such recruitment was obvious in most recorded slices (*n* = 11 slices/5 tree shrews with TBS; *n* = 7 slices/3 tree shrews with weak TBS), but some slices did not show any recruitment (*n* = 2 slices/2 tree shrews with weak TBS). Such recruited fEPSP could be accounted for in all slices and only distributed in scattered channels on the edge of the active area. However, most of the edge channels could not be activated by TBS or weak TBS induction, indicating that the recruitment is unlikely owing to changed stimulation intensities or unstable recordings.

### (e) The involvement of NMDAR, L-type Ca^2+^ channel and adenylyl cyclase 1 in the induction of LTP

**Figure 6.**
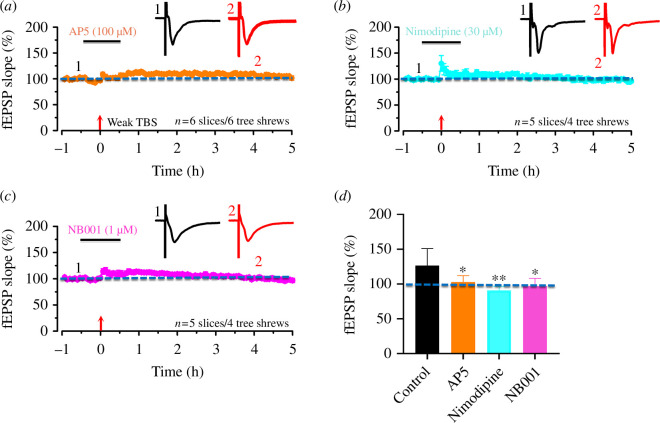
Weak TBS-induced the post-LTP depends on the activation of NMDA receptor, L-type voltage-gated calcium channels and adenylyl cyclase 1 in the ACC of tree shrew. (*a*) The final averaged slope for all 97 active channels from 6 slices of 6 tree shrews was 105.1 ± 2.0% of the baseline at 5 h after weak TBS application in AP5 (100 μM). (*b*) The final averaged slope for all 90 active channels from 5 slices of 4 tree shrews was 102.3 ± 4.8% of the baseline at 5 h after weak TBS application in nimodipine (30 μM). (*c*) The final averaged slope for all 150 active channels from 5 slices of 4 tree shrews was 97.1 ± 4.7% of the baseline at 5 h after weak TBS application in NB001 (1 μM). (*d*) Bar histogram summarizing quantified data within the last 30 mins of the 5 h recording. Unpaired Student’s *t*‐test, *p < 0.05, **p < 0.01. Arrows in (*a–c*) indicate the starting point of weak TBS application. Error bars in (*a–d*) represent s.e.m.

The direction of the plasticity is largely controlled by the kinetics and amount of Ca^2+^ influx through the NMDA receptors on the synapses [42]. The L-type Ca^2+^ channel takes an important role in mediating Ca^2+^ influx in the ACC [43], where calmodulin-stimulated AC1 is critical for LTP. To test whether NMDA receptor, L-type Ca^2+^ channels and AC1 are required for the induction of cingulate network L-LTP in tree shrew, we applied the NMDA receptor, L-type Ca^2+^ channels and AC1 antagonist AP5, nimodipine and NB001, respectively, both before and after TBS or weak TBS protocol. After the AP5 (100 μM) application, the baseline remains stable, indicating that the AP5 does not influence the basic transmission in tree shrew. The slope of all fEPSP showed little potentiation (115.4 ± 1.1% of baseline for 5 slices/4 tree shrews) with TBS and no potentiation (106.0 ± 0.5% of baseline for 6 slices/6 tree shrews, figure 6*a,d*) with weak TBS. With the same tendency, the nimodipine does not influence the basic transmission in tree shrew. The slope of fEPSP still showed potentiation (123.5 ± 0.7% of baseline for 7 slices/5 tree shrews) with TBS and no potentiation (102.3 ± 4.8% of baseline for 5 slices/4 tree shrews, *p* < 0.05, figure 6*b,d*) with weak TBS. For the NB001 application, the baseline remains stable, indicating that AC1 does not influence the basic transmission in tree shrew. The slope of all fEPSP showed little potentiation (116.6 ± 11.1% of baseline for 6 slices/6 tree shrews) with TBS and no potentiation (102.3 ± 2.0% of baseline for 5 slices/4 tree shrews, figure 6*c,d*) with weak TBS. The results showed that AP5, nimodipine and NB001 all have an effective, although not complete, attenuation of synaptic potentiation in tree shrew with TBS and weak TBS (both *p* < 0.01 and *p* < 0.05 in comparison with the control group, unpaired *t*‐test, data not shown). The results demonstrate that NMDA receptor, L-type Ca^2+^ channels and AC1 are important for LTP induction in tree shrew.

## 4. Discussion

In the present study, we demonstrate for the first time that TBS induced a long-lasting LTP in the ACC of adult tree shrews. The amplitude of LTP in the adult tree shrew is significantly greater than that of adult mice, suggesting that cortical LTP plays more important roles in primate-like animals.Tree shrew is an excellent model for the study of cortical transmission and plasticity. Similar to the ACC of adult mice, excitatory transmission is mostly mediated by glutamate, and postsynaptic AMPA receptors contribute to basal synaptic transmission.

The MED64 system used in the present study allows us to investigate the ACC circuit at the same time. Our pharmacological results confirm the findings from our previous report that glutamate is the major excitatory transmitter [44]. Both AMPA and Kainic acid (KA) receptors contribute to synaptic transmission, and it is quite likely that excitatory synapses are heterogenous. Our previous studies found that there are both pure AMPA receptor-containing synapses and synapses containing a moxture of AMPA and KA receptors in the ACC of adult mice [45,46]. The nerve projections from different regions of cortical and subcortical areas to the ACC are one of the reasons for such heterogeneity. In addition, silent synapses have been reported in adult cortex of mice [47–49]. In the present study, we found that some silent responses in the ACC can be recruited by LTP. Furthermore, this recruitment is long-lasting and persists for at least 5 h. Our results strongly suggest that silent glutamatergic synapses may exist in the brain of adult primate-like animals, and the recruitment of these silent synapses could contribute to learning and memory.

LTP is the popular cellular model for investigating molecular mechanisms for synaptic changes under physiological and pathological conditions [20,21,50–52]. In addition to commonly used mice, rats and guinea pigs, LTP has also been investigated in rabbit [53,54], cat [55,56] and pig [57,58]. Although ACC has been thought to be important for many higher-order brain functions, previous studies of LTP in the ACC have only been carried out in rats and mice [20,21,52]. Therefore, our work presents an important confirmation for the existence of cortical LTP in primate-like animals’ brains.

It is difficult to obtain a stable recording of lasting LTP in adult neurons using the whole-cell patch clamp recording method. The MED64 system has overcome this problem. As we have shown before in adult mice and in the current work in tree shrew, TBS can induce long-lasting LTP in the ACC for at least 3–6 h [23,33]. In the ACC of tree shrew, we found that TBS induced long-lasting LTP for at least 5 h. More importantly, the magnitude of LTP is much greater than that seen in mice, supporting the fact that tree shrew are much more intelligent and smarter than mice. According to this finding, it is quite likely that LTP in adult monkeys or human brains may be even more important.

The intracellular mechanism for ACC LTP has been well investigated in adult mice [20]. In the present study, we found that tree shrews' LTP requires the activation of NMDARs, including GluN2B-containing NMDARs. This finding is similar to a previous report in adult mice [22]. In addition, we found that L-type voltage-dependent calcium channels (L-VDCCs) also contribute to LTP, indicating that there are at least two possible mechanisms for triggering ACC LTP in the tree shrew. These results are consistent with previous findings in adult mice, suggesting that LTP in these two species shares similar mechanisms [20]. Calcium-stimulated AC1 has been reported to contribute to ACC LTP in adult mice, both in the genetic deletion of the AC1 gene and a selective pharmacological inhibitor NB001 [59–62]. In this study, we found that NB001 blocked LTP in the tree shrew ACC, indicating that NMDA (GluN2B) receptor-AC1-cAMP plays an important role in ACC LTP of the adult tree shrew. Considering the important roles of AC1 in chronic pain and pain-related emotional fear and anxiety [20,21,52], it is likely that it could play important roles in chronic pain and emotional disorders in primates and humans.

In summary, our present results provide the first evidence to demonstrate the field excitatory postsynaptic potentials, induced the late-phase LTP and spatial propagation in the ACC of tree shrew. We also tested the mechanism of LTP regulation and found that the AC-cAMP signal cascade takes an important role in this process. This provides the foundation of the regulation mechanism of LTP in primates for our future research.

## Data Availability

All data needed to evaluate the conclusions in the paper are present in the paper and/or the Supplementary Materials [63].
